# Annotations

**Published:** 1887-10-01

**Authors:** 


### Annotations.

Microbes.

For thirty years those wonderful little creatures which can only be seen and studied under the higher powers of the microscope have claimed the best powers of some of the most laborious scientific workers of the period. To the mind of science no creatures endowed with life are insignificant, nor is anything they can do astonishing. To the uninstructed the fact that a million microbes may be destroyed by the boiling of a teacupful of water, is of a nature to make him regard them with contempt. But let such a man become the subject of typhoid or other microbial fever, and he is in a fair way of giving to his despised enemies an ample revenge. The darkness is rapidly clearing away in the "germ" world. Like the spirits which used to haunt the midnight hours in the days of our forefathers, the little creatures are not all dangerous and malignant. Many of them appear to be quite harmless under ordinary circumstances, and it may be that they will all prove to be so when we shall have learnt to manage ourselves and our health better. In a recently published paper, Mr. Percy Faraday Frankland gives the results of experiments in this field of science. According to these, microbes may generally be found in atmospheric air, but their number will vary with the quality, temperature, and altitude of the air. In the cold months the microbes are fewest, and conversely, in the hottest months their numbers reach the maximum. Thus, in a given volume of air, equal to about two gallons (ten litres), collected on the top of the Science and Art Department buildings at South Kensington, about seventy feet from the ground, there were found in January only four microbes, whilst in July the same volume at the same place yielded sixty-three, and in August a hundred and five; the number afterwards gradually falling each month, until in December twenty only were found. Two volumes of air examined at the foot of Primrose Hill yielded twenty-four microbes, whilst the same volume at the top gave nine. At the base of the tower of Norwich Cathedral eighteen were found; on the top of the tower, 180 feet in height, only half that number. In a third-class railway carriage with four persons in it, 395 organisms fell on a square foot in one minute; in the same carriage at a later period, and with ten persons in it, there were no less than 3,120. The main point for practical persons is to ascertain the significance of these undoubted facts; and Professor Burdon Sanderson sums up in a few words their bearing on every-day life. He says:—" Considering that we know the living dust of the air *does* contain organisms which are capable of producing putrefaction and inflammation in wounds . . . . and that it *may* contain the distinctive or specific poisons of particular diseases . . . . therefore . . . . it behoves everyone to be as careful as possible to maintain the air as free as possible from these minute organisms, not because they are all dangerous, but because we do not know where the danger lurks."

A Strange Locum Tenens.

Many a doctor fears to take his much needed annual holiday because of the difficulty of the *locum tenens* question. He remembers too well his past experiences: how one *locum* broke the knees of his new five-year-old mare, another thrashed the medicine-boy, and was haled by an indignant parent before the local magistrate, whilst a third gave such mortal offence to his best chronic patient that she there and then sent for Dr. Brown over the way, and has ever since paid that greedy and triumphant practitioner at least fifty pounds a year. The unhappy medicus thinks no one is so hardly dealt with as himself in this matter of holidays; but the Rev. Dr. Jessop, in the August number of the *Nineteenth Century*, tells a story of a clerical *locum tenens* which makes all stories of medical temporary substitutes "pale their ineffectual fires." The rector of Cotton-in-the-Brake was a courtly man of fifty, who, having received an accession of fortune, resolved to marry. But how was he to find a man among the perambulating clergy fit to stand in his dignified shoes? He did, however, as he fondly hoped, find the right man at last. The Rev. Mr. Connor was a scholarly, well-dressed, and evidently accomplished gentleman, who spoke of Mrs. Connor with respectful confidence and affection, who had been married ten years, and who made no difficulties, except that he thought the stables might be inconveniently small. With a mind relieved and a blissful honeymoon before him, the rector set out for Nice; and in due time the Rev. Mr. Connor arrived at the rectory. Mrs. Connor came too, with *fourteen brindled bull-dogs*. That was her specialty, and she gave all her mind to keeping the breed pure. Next appeared seven pupils, the rejected of the public schools. Mrs. Connor kept no female servants. Not a woman dared pass the rectory gates. She had a man cook and *men housemaids*. The bull-dogs would prowl about the neighbourhood sniffing, growling, turning their hideous bloodshot eyes upon you, undecided whether or not to tear you limb from limb. Sometimes there were rumours of horrible fights, and no one dared separate the brutes except Mrs. Connor, who always kept a bar of iron with a wooden handle red-hot in the kitchen fire, for that purpose. As Dr. Jessop adds, "Imagine the condition of that newly furnished parsonage when the poor rector came back to his home!"

Compulsory Notification of Infectious Diseases.

Wherever scientific, sanitary, or medical people meet in council there, almost certainly, will the compulsory notification of infectious diseases be a subject of discussion. It need not be questioned that the public well-being would be furthered by the general practice of notifying to the Registrar every such case. Nobody doubts that, unless it be some faddist of the anti-vaccination or Shaker type. The really important question is—who is to be the notifier? An unthinking public and a lazy and greedy officialism see one trustworthy notifier ready to hand, and at once, like the monks with the Jackdaw of Rheims, they unitedly, and with equal disregard of grammar, call out, "that's him"—"him" in this case being the family doctor. Now we venture to say, and with emphasis—" that's *not* him." The family doctor should no more be made responsible for the notification of infectious diseases which come under his care than the family tailor for the notification to the Registrar of every new coat he supplies to a customer. Every man must be responsible for the members of his own family, and if the State desires any knowledge of that family, or the performance of anything which it chooses to make a duty, then the proper person to apply to is the head of the house. To attempt to saddle the doctor with such responsibility can have no recommendation but that of convenience, and the members of the medical profession will be well advised to resist to the utmost a regulation which, while it may suit the public, may entail upon them untold worries and very frequently considerable pecuniary loss.

